# Co-Expression Network and Machine Learning Analysis of Transcriptomics Data Identifies Distinct Gene Signatures and Pathways in Lesional and Non-Lesional Atopic Dermatitis

**DOI:** 10.3390/jpm14090960

**Published:** 2024-09-10

**Authors:** Eskezeia Y. Dessie, Lili Ding, Latha Satish, Tesfaye B. Mersha

**Affiliations:** 1 Division of Asthma Research, Cincinnati Children’s Hospital Medical Center, Department of Pediatrics, University of Cincinnati College of Medicine, 3333 Burnet Avenue, Cincinnati, OH 45229-3039, USA; eskezeia.dessie@cchmc.org (E.Y.D.); latha.satish@cchmc.org (L.S.); 2 Division of Biostatistics and Epidemiology, Cincinnati Children’s Hospital Medical Center, Department of Pediatrics, University of Cincinnati College of Medicine, 3333 Burnet Avenue, Cincinnati, OH 45229-3039, USA; lili.ding@cchmc.org

**Keywords:** atopic dermatitis (AD), weighted co-expression network analysis, pathway expression analysis, machine learning, diagnostic biomarkers

## Abstract

Background: Atopic dermatitis (AD) is a common inflammatory skin condition with complex origins. Current treatments often yield suboptimal results due to an incomplete understanding of its underlying mechanisms. This study aimed to identify pathway and gene signatures that distinguish between lesional AD, non-lesional AD, and healthy skin. Method: We conducted differential gene expression and co-expression network analyses to identify differentially co-expressed genes (DCEGs) in lesional AD vs. healthy skin, lesional vs. non-lesional AD, and non-lesional AD vs. healthy skin. Modules associated with lesional and non-lesional AD were identified based on the correlation coefficients between module eigengenes and clinical phenotypes (|R| ≥ 0.5, *p*-value < 0.05). Subsequently, we employed Ingenuity Pathway Analysis (IPA) on the identified DCEGs, followed by machine learning (ML) analysis within the pathway expression framework. The ML analysis of pathway expressions, selected by IPA and derived from gene expression data, identified relevant pathway signatures, which were validated using an independent dataset and correlated with AD severity measures (EASI and SCORAD). Results: We identified 975, 441, and 40 DCEGs in lesional vs. healthy skin, lesional vs. non-lesional, and non-lesional vs. healthy skin, respectively. IPA and ML analyses revealed 25 relevant pathway signatures, including wound healing, glucocorticoid receptor signaling, and S100 gene family signaling pathways. Validation confirmed the significance of 10 pathway signatures, which were correlated with the AD severity measures. DCEGs such as MMP12 and S100A8 demonstrated high diagnostic efficacy (AUC > 0.70) in both the discovery and validation datasets. Conclusions: Differential gene expression, co-expression networks and ML analyses of pathway expression have unveiled relevant pathways and gene signatures that distinguish between lesional, non-lesional, and healthy skin, providing valuable insights into AD pathogenesis.

## 1. Introduction

Atopic dermatitis (AD) is a multifactorial and complex skin disease that affects approximately 30% of children [1] and 3–5% of adults globally [2]. AD presents as a highly heterogeneous phenotype and can manifest in both lesional and non-lesional skin forms [3]. While lesional AD skin is characterized by pronounced inflammation, increased water loss, and compromised barrier function, non-lesional AD skin appears visually healthy but harbors subtle features typical of diseased skin [4,5,6]. Non-lesional areas may play a role in the development of active lesions and increased skin permeability over time. Notably, the trans-epidermal water loss (TEWL) values in non-lesional skin are relatively higher than those in healthy skin, although the differences are not significant [7]. In contrast, lesional AD skin consistently shows significantly elevated TEWL values compared to both non-lesional and healthy skin [8]. Consequently, numerous studies have aimed to discriminate between lesional AD and non-lesional skin [9,10].

In recent years, with the development of high-throughput omics technologies, large-scale transcriptomic AD data can help to understand the underlying molecular mechanisms of AD [11]. Several studies have explored potential biomarkers by comparing gene expression patterns in lesional and non-lesional skin of AD patients. Dyjack et al. investigated skin tape strip samples and found overexpression of IL-13, IL-4R, CCL22, and CCR4 in severe AD [9]. Using transcriptome data from non-lesional skin allowed stratification of patients based on disease severity. Several biomarkers distinguishing between lesional and non-lesional AD samples have also been proposed [10,12,13]. In addition, dysregulated genes in enriched pathways, including atherosclerosis signaling [14], have been implicated in AD pathogenesis, reflecting immune and inflammatory processes [15]. Recent research highlights the activation of the TH22, TH17/IL-23, and TH1 pathways in AD skin subtyping [16].

Despite recent breakthroughs, biological therapies for diagnosing non-lesional and lesional AD remain less understood. AD diagnosis currently relies on patient history and visual assessment, with management involving topical treatments (moisturizers, corticosteroids, or calcineurin inhibitors) that lack specificity for AD [17]. While clinical manifestations of AD skin have been well documented, the molecular distinctions between lesional and non-lesional AD skin remain incompletely studied. Identifying pathways involved in the transition from non-lesional to lesional AD skin is crucial.

The recent literature explored ML models for biomarker discovery in AD, leveraging omics data. Martínez et al. developed ML methods to examine four skin diseases, including AD, using gene expression data and differentiating non-lesional samples from normal skin [10]. Zhong et al. used the LASSO method to discover three biomarkers distinguishing lesional from non-lesional AD skin [18]. A recent study used random forest (RF) to identify two AD endotypes, including eosinophil low and eosinophil high, using blood transcriptome data [12]. Several studies have conducted biological pathway analysis using biological relationships between genes, usually using enrichment analysis. However, ML- analysis of expression data at the pathway level or pathway expression analysis to distinguish lesional and non-lesional skin in AD has not been explored. Pathway expression analysis leverages the biological interconnectedness of genes to convert gene expression data into pathway expression data, facilitating the identification of pathway signatures [19,20].

In our study, we used an integrated analysis, including differential gene expression analysis, co-expression network analysis, and IPA and ML analysis of pathway expression to select pathway and gene signatures that discriminate three comparisons: lesional AD vs. heathy skin, lesional vs. non-lesional AD and non-lesional AD vs. healthy skin. We used gene expression datasets, including GSE121212, GSE107361 and GSE130588, from the Gene Expression Omnibus (GEO) database. Potential DCEGs in three pairwise group comparisons were first identified. Then, we identified the underlaying major significant pathways associated with the DCEGs and constructed pathway expression data matrices using the annotated DCEGs in the pathways. The RF method and importance score were used to identify and prioritize pathways that discriminate lesional, non-lesional AD and healthy skin in the GSE121212 dataset. The GSE107361 and GSE130588 datasets were used to validate the pathways and gene signatures associated with lesional, non-lesional and AD severity indices, including EASI and SCORAD. Overall, our integrative analyses were able to provide novel insights to identify important pathways and gene signatures that discriminate lesional vs. healthy, lesional vs. non-lesional and non-lesional vs. healthy skin and provide potential therapeutic targets.

## 2. Materials and Methods

### 2.1. Dataset Used in the Study

In our study, we harnessed gene expression data and clinical information from publicly available databases to explore the transcriptomic landscape of AD. Our inclusion criteria included any dataset that (1) studies Homo sapiens, (2) has a sample size ≥ 50, and (3) contains the gene expression profiles of lesional, non-lesional and healthy skin samples. Using these inclusion criteria, we found three relevant Gene Expression Omnibus (GEO) datasets: GSE121212, GSE107361, and GSE130588 to understand the molecular underpinnings of lesional and non-lesional AD. We leveraged the RNA-sequencing (RNA-seq) GSE121212 dataset from 92 skin samples, including 27 non-lesional, 27 lesional AD and 38 healthy skins, as the discovery dataset to identify genes and pathway signatures associated with lesional and non-lesional AD. The details of the subject recruitment and study procedure were described in previous studies [21,22]. Dermatologists diagnosed AD based on standardized criteria, and high-depth RNA-seq allowed us to unravel the disease mechanisms. The other two datasets, GSE107361 and GSE130588, which consist of gene expression profiles and the corresponding clinical data, were used for the validation of the results. The GSE107361 dataset consists of 39 healthy, 40 non-lesional and 29 lesional AD skin samples with microarray gene expression profiles. This dataset was preprocessed and normalized in the original study [23]. The GSE130588 dataset consists of 51 lesional, 41 non-lesional AD and 20 healthy skin biopsy specimens along with the disease severity indices EASI and SCORAD collected at baseline in the original study. Data preprocessing and normalization were described in a previous study [24]. The GSE130588 dataset was used to examine the association of the pathway expression and disease severity index measures. A summary of the datasets used in our study is shown in Supplementary Table S1.

### 2.2. Differential Expression Genes (DEGs) Analysis

In our study, we applied rigorous data preprocessing steps to the RNA-seq discovery data. Low-abundance genes (<10 reads) were removed to enhance the robustness of the RNA-seq discovery data [25]. Using the DESeq2 [26] method with variance stabilizing transformation, we performed pairwise differential expression analysis across different groups (lesional AD vs. healthy, lesional vs. non-lesional AD, and non-lesional AD vs. healthy). Genes with adjusted *p*-values (Benjamini–Hochberg) < 0.05 and an absolute log2 fold change > 1 were deemed significant.

### 2.3. Weighted Gene Co-Expression Network Analysis (WGCNA)

In our study, we focused on identifying co-expressed genes alongside individual differentially expressed genes (DEGs) in AD. By calculating the coefficient of variation (CV) for each gene in the variance-stabilized and normalized RNA-seq data, we pinpointed hypervariable genes (CV > 15%) across three pairwise group comparisons: healthy vs. lesional, healthy vs. non-lesional, and lesional vs. non-lesional in the discovery cohort [26]. Next, we conducted gene co-expression network analysis using the “WGCNA” package [27]. Initially, we constructed a similarity matrix based on the pairwise correlation $S_{ij}$ = $cor\;(x_{i},\;x_{j}$), where $x_{i}$ and $x_{j}$ represent the ith row and the jth row of hypervariable gene normalized RNA-seq data matrix X, respectively, and then we transformed the similarity matrix into an adjacency matrix, denoted by $A_{ij}={\left|cor\;(x_{i},\;x_{j})\right|}^{\beta }$, where *β* represents the suitable soft-thresholding power (ranges from 1–20) and the suitable powers were estimated using the index value in the discovery dataset of each comparison group based on the pick Soft Threshold function [27]. Next, we transformed the adjacency gene network into a topological overlap matrix (TOM) and corresponding dissimilarity (1-TOM) matrix. The WGCNA used hierarchical clustering to identify gene modules/clusters of densely interconnected genes in many samples and color to denote the modules. The minimum number of genes in each module was set to 50 [28].

### 2.4. Identifying Modules Correlated with Lesional and Non-Lesional Skin

Here, we employed the Spearman correlation coefficient between the clinical phenotypes (lesional AD, non-lesional AD, and healthy skin) and the module eigengenes (MEs), the principal component of a module, to identify crucial gene modules. Genes in the modules correlated with the clinical phenotypes (absolute Spearman correlation coefficient (|R|) > 0.5 and *p*-value < 0.05) and DEGs obtained from differential analysis were then overlapped, pinpointing differentially co-expressed genes (DCEGs) or hub genes across three group comparisons.

### 2.5. Functional Enrichment Analysis

The pathway enrichment analyses were conducted using the Ingenuity Pathway Analysis software (IPA, QIAGEN, Redwood City, CA, USA) [29] to explore the pathway behaviors of genes within modules correlated with the AD clinical phenotypes and DEGs. For this analysis, we focused on gene signatures overlapping between DEGs meeting specific criteria: absolute log2 fold-change values > 1 and adjusted *p*-value < 0.05 and DCEGs in modules with an absolute Spearman correlation coefficient (|R|) > 0.5 and *p*-value < 0.05. IPA pathways with an adjusted *p*-value < 0.05 were considered in pathway expression analysis below.

### 2.6. Machine Learning Analysis of Pathway Expression and Statistical Analyses

In this study, we utilized pathway expression analysis instead of gene expression level analysis approach [19,20] to identify key pathways. For the pathway expression analysis, we first constructed a pathway expression matrix from the gene expression matrix in the discovery and validation datasets. The general transformation of the gene expression matrix into the pathway expression matrix is shown in Supplementary Figure S1. The pathway expression matrix was then defined as the average expression level of all the annotated genes for each subject and each significant pathway. These pathway expression matrices served as features for the supervised RF analysis, and relevant pathways that discriminated between lesional, non-lesional, and healthy skin were identified. Relevant pathways with an importance score, measured by a mean decrease in accuracy > 4 in classifying the AD clinical phenotype, including lesional, non-lesional AD and healthy skin, were selected using the RandomForests R package. The heatmaps of the AD clinical phenotype discriminatory pathways were visualized using the pheatmap R package. The mean pathway expression across the pairwise comparisons including lesional vs. healthy, non-lesional vs. lesional, and non-lesional vs. healthy skin were compared using *t*-tests in the discovery and validation normalized gene expression datasets. Pearson correlation (R) analysis was applied to examine the correlation between the pathway expression data and the numerical AD disease severity measures, including EASI and SCORAD, in the validation dataset. An area under the receiver operating characteristics (ROC) curve using a logistic regression model was used for assessing the diagnostic efficiency of the pathway expression and gene signatures in discriminating lesional vs. healthy, lesional vs. non-lesional and non-lesional vs. healthy skin in the discovery and validation datasets. All the statistical analyses were conducted using R v4.3.2. A *p*-value < 0.05 was used to define statistical significance, unless otherwise stated.

## 3. Results

### 3.1. Identification of Differentially Expressed Genes in Lesional and Non-Lesional AD Skin

**Figure 1:**
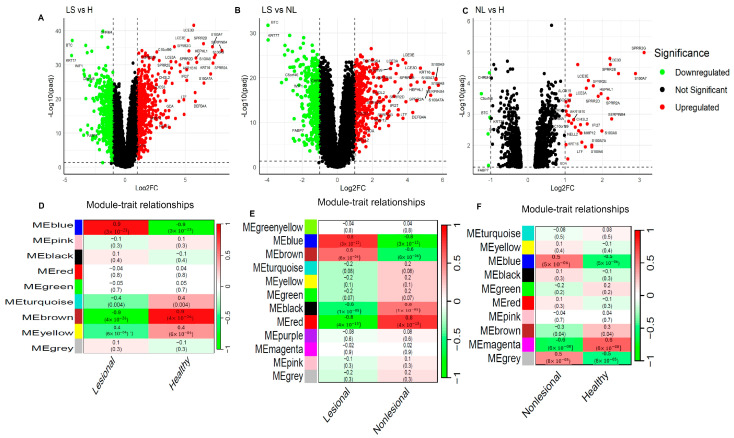
Identification of lesional- and non-lesional-associated genes in the discovery dataset. (**A**–**C**) Volcano plot indicating 1243 differentially expressed genes (DEGs with 544 up- and 699 down-regulated) in lesional AD compared to healthy skin, 801 DEGs (355 up- and 446 down-regulated) in lesional compared to non-lesional AD skin and, 42 DEGs (36 up- and 6 down-regulated) in non-lesional AD compared to healthy skin, respectively, in the discovery RNA-seq dataset. A total of 33 common DEGs were identified across three comparisons, as highlighted in the volcano plots. Red dots indicate genes with an adjusted *p*-value < 0.05 and log2 fold change (log2FC) > 1; green dots indicate genes with an adjusted *p*-value < 0.05 and log2FC < 1; DEGs, differentially expressed genes. (**D**) The correlation between the module eigengenes and the clinical phenotype: lesional AD vs. healthy skin. (**E**) The correlation between the module eigengenes and the clinical phenotype: lesional vs. non-lesional AD skin. (**F**) The correlation between the module eigengenes and the clinical phenotype: non-lesional vs. healthy skin. Key module genes associated with lesional and non-lesional skin were selected with the following criteria: absolute correlation coefficient, |R| > 0.5 and *p*-value < 0.05. LS-lesional, NL-non-lesional, H-healthy skin, and R-Spearman’s correlation coefficient.

Differential gene expression analysis of the lesional (*n* = 27), non-lesional (*n* = 27) AD and healthy control (*n* = 38) samples revealed 1243 DEGs (544 up- and 699 down-regulated) in lesional vs. healthy, 801 DEGs (355 up- and 446 down-regulated) in lesional vs. non-lesional AD, and 42 DEGs (36 up- and 6 down-regulated) in non-lesional AD vs. healthy skin (Figure 1A–C) in the discovery dataset. Notably, we identified 33 common DEGs (27 up-regulated and 6 down-regulated genes) across three pairwise comparisons, as shown in Figure 1A–C and Supplementary Figure S2A. Unsupervised cluster analysis of these DEGs in the three comparison groups successfully discriminated between lesional, non-lesional, and healthy skin (Supplementary Figure S2B). The observed higher absolute log2 fold change in lesional vs. healthy skin, compared to lesional vs. non-lesional and non-lesional vs. healthy skin (as shown in Supplementary Figure S2B), suggests that several genes are significantly dysregulated in lesional AD. These include up-regulated genes such as SERPINB4, S100A9, S100A8, SPRR2A, S100A7, S100A7A, SPRR2B, KRT16, HEPHL1, and DEFB4A, as well as down-regulated genes such as KRT77, BTC, WIF1, FABP7, CHRM4, ALOX15, SOCS3, NELL2, TMPRSS4, and MMP12), which may enhance our understanding of the AD disease mechanisms and contribute to personalized medicine.

### 3.2. Identification of Differential Co-Expression Modules Associated with Lesional and Non-Lesional AD

To explore the similarity and heterogeneity of biological molecules in lesional and non-lesional AD, we first identified 4905 hypervariable genes using coefficient of variation analysis across three pairwise comparisons in the discovery dataset. Next, co-expression network analyses of these 4905 hypervariable genes were conducted across the three pairwise comparisons using appropriate soft threshold powers (β = 5, 10, and 5, with scale-free R^2^ ≥ 0.85) for the three comparisons (Supplementary Figure S3A–C) to investigate their gene regulatory networks. We identified a total of 9, 12, and 10 co-expression modules in lesional vs. healthy, lesional vs. non-lesional, and non-lesional vs. healthy comparisons (Supplementary Figure S4A–C), respectively. Of these, the key gene modules correlated with lesional and non-lesional AD (|R| > 0.5 and *p*-value < 0.05) were selected for further analysis (Figure 1D–F and Supplementary Table S2). For the lesional AD and healthy skin comparison, the blue module with 1171 genes is positively correlated with lesional AD, while the brown module with 637 genes is negatively correlated with lesional AD (Figure 1D). In the lesional and non-lesional AD comparison, the blue module (601 genes) and brown module (459 genes) are positively correlated with lesional AD, whereas the black module (236 genes) and red module (312 genes) are negatively correlated with lesional AD (Figure 1E). For the non-lesional AD and healthy skin comparison, the blue module (765 genes) is positively correlated and the magenta module (68 genes) is negatively correlated with non-lesional AD (Figure 1F).

After identifying the co-expressed genes/module genes, we conducted an overlapping analysis between the DEGs and the module genes in three pairwise comparisons to screen DCEGs in lesional and non-lesional AD (Supplementary Table S2). For lesional AD and healthy skin, we identified 338 DCEGs in the blue module and 244 DCEGs in the brown module. For lesional AD and non-lesional AD, we identified 240 DCEGs in the blue module, 32 DCEGs in the brown module, 73 DCEGs in the black module, and 96 DCEGs in the red module. For non-lesional AD and healthy skin, we identified 35 DCEGs in the blue module and 5 in the magenta module.

Overall, our analyses revealed 975, 441, and 40 DCEGs in the lesional vs. healthy, lesional vs. non-lesional, and non-lesional vs. healthy skin comparisons, respectively. Notably, there were more DCEGs in lesional AD vs. healthy skin, suggesting that more gene regulatory networks are involved in lesional AD.

### 3.3. Functional Enrichment Analysis of the DCEGs in Lesional and Non-Lesional Correlated Modules

To further understand the biological function of DCEGs within modules correlated with non-lesional and lesional AD, we performed a canonical pathway enrichment analysis. We identified a total of 108 over-represented pathways (*p*-value < 0.05, Supplementary Tables S3–S7). The 338 DCEGs in the blue module derived from lesional AD and healthy skin are involved in several pathways, including granulocyte adhesion and diapedesis, atherosclerosis signaling, granulocyte adhesion and diapedesis, pathogen-induced cytokine storm signaling pathway, interferon signaling and S100 family signaling pathways (Supplementary Table S3). The 244 DCEGs in the brown module derived from lesional and healthy skin are enriched in 10 pathways, including the 3 most significant pathways, the role of osteoblasts in rheumatoid arthritis signaling, neurovascular coupling signaling and iron homeostasis signaling (Supplementary Table S4). The 240 DECGs in the blue module derived from lesional and non-lesional AD are enriched in 51 pathways, with the 5 most significant pathways included atherosclerosis signaling, interferon signaling, S100 family signaling, IL-17 signaling, and differential regulation of cytokine production in intestinal epithelial cells by IL-17A and IL-17F (Table S5). The 32 DCEGs in the brown module derived from lesional and non-lesional are involved in several pathways, including granulocyte adhesion and diapedesis, agranulocyte adhesion and diapedesis, atherosclerosis signaling, and pathogenesis of multiple sclerosis (Supplementary Table S6). The 35 DCEGs in the blue module derived from non-lesional and healthy skin are enriched in 9 pathways, including the IL-13 signaling pathway, pathogen-induced cytokine storm signaling pathway, and granulocyte adhesion and diapedesis pathways (Supplementary Table S7).

### 3.4. Identification of Lesional and Non-Lesional AD Discriminatory Pathway Signatures

**Figure 2:**
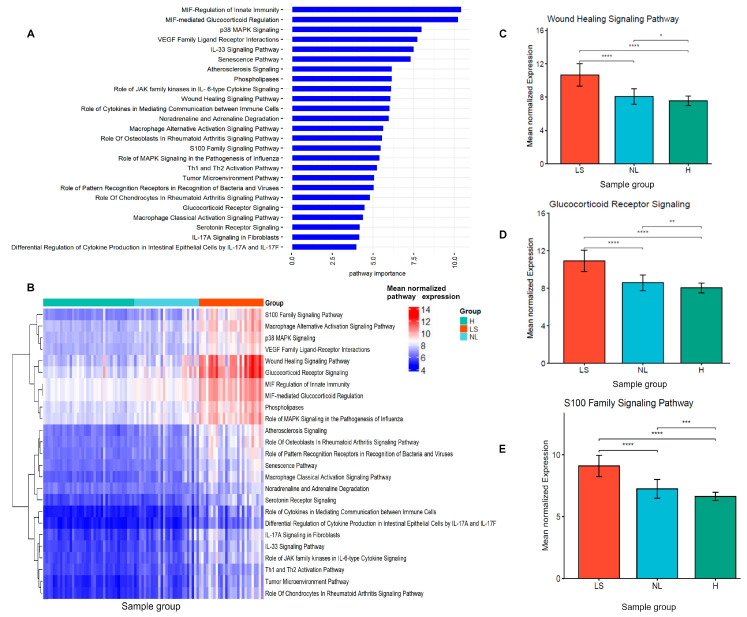
Identification of the pathway signatures discriminating lesional AD, non-lesional AD and healthy skin in the discovery dataset. (**A**) Most important pathway signatures selected by the random forest method. (**B**) The heatmap showing the mean normalized expression level of 25 pathway signatures among three groups. (**C**–**E**) Comparison of the mean normalized expression level of the pathway signatures in three pairwise comparisons. The significance levels of the comparison pathway mean differential modulation across the pairwise group comparisons are indicated by the stars (**** *p* < 0.0001, *** *p* < 0.001, ** *p* < 0.01, * *p* < 0.05). LS-lesional, NL-non-lesional, H-healthy skin.

As shown in the functional enrichment analysis, we found several significant pathways enriched in lesional and non-lesional AD. To prioritize and identify important pathways that differentiate lesional and non-lesional AD, we first transformed the gene expression data into pathway expression-level data by calculating the average gene expression levels within each of the 108 enriched pathways. Then, we used RF algorithms and selected 25 pathway expression signatures based on their discriminatory importance for lesional, non-lesional AD, and healthy skin (Figure 2A,B). These pathways exhibited differential modulation across the comparisons, with higher expression profiles in lesional AD compared to non-lesional AD and healthy skin (Figure 2C–E and Supplementary Figure S5).

### 3.5. Enriched Pathway Signatures Were Correlated with AD Clinical Severity Measures

**Figure 3:**
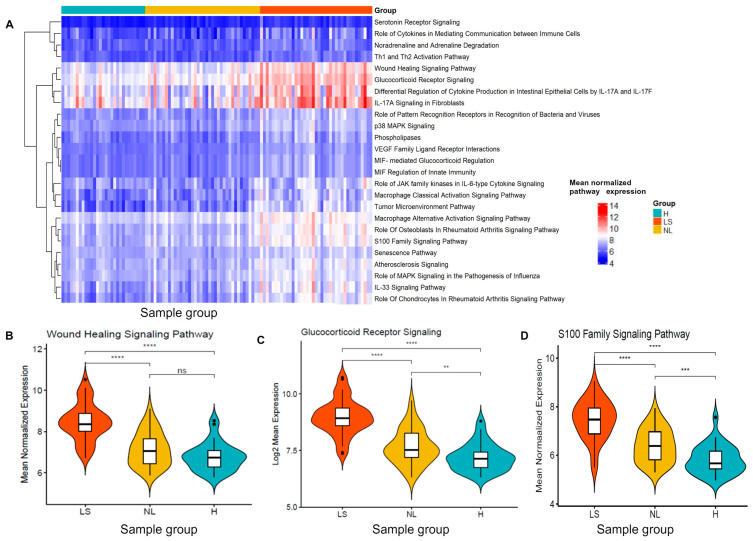
Validation of the pathway signatures in discriminating lesional, non-lesional and healthy skin in the independent validation dataset. (**A**) The heatmap showing the mean normalized expression level of the 25 pathway signatures among three groups. (**B**–**D**) Comparison of the mean normalized expression level of the pathway signatures in three pairwise comparisons. The significance levels of the comparison pathway mean differential modulation across pairwise group comparisons were indicated by the stars (**** *p* < 0.0001, *** *p* < 0.001, ** *p* < 0.01, ns: not significant). LS-lesional, NL-non-lesional, H-healthy skin.

**Figure 4:**
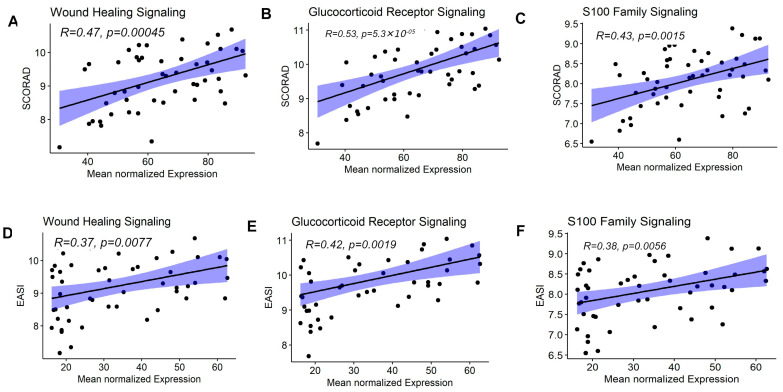
Validation of the association of the pathway mean expression levels and the disease severity measures, including the SCORAD and EASI, in lesional AD skin. (**A**) The correlation between the mean normalized expression of genes in the wound-healing signaling pathway and the SCORAD score. (**B**) The correlation between the mean normalized expression of genes in the glucocorticoid receptor signaling pathway and the SCORAD score. (**C**) The correlation between the mean normalized expression of genes in the S100 family signaling pathway and the SCORAD score. (**D**) The correlation between the mean normalized expression of genes in the wound-healing signaling pathway and the EASI score. (**E**) The correlation between the mean normalized expression of genes in the glucocorticoid receptor signaling pathway and the EASI score. (**F**) The correlation between the mean normalized expression of genes in the S100 family signaling pathway and the EASI score.

After identifying the pathway expression signatures, we validated the pathway expression signatures using independent datasets. Specifically, we analyzed the GSE107361 dataset to confirm the differential modulation of ten pathway expression signatures across lesional, non-lesional, and healthy skin. Notably, ten pathway expression signatures, including wound healing, glucocorticoid receptor signaling and S100 family signaling, were differentially modulated among lesional, non-lesional AD and healthy skin (Figure 3A–D and Supplementary Figure S6). Genes implicated in wound healing (CXCL8, HBEGF, IL36A, IL36G, KRT16, KRT17, KRT6A, KRT6B, KRT6C, MMP1 and PGF), genes implicated in glucocorticoid receptor signaling (CCL2, CXCL8, IL4R, KRT16, KRT17, KRT6A, KRT6B, KRT6C, MMP1, PLA2G2F, PLA2G and SELE) and genes implicated in S100 family signaling (CCL20, CCR7, CXCL8, MMP1, MMP12, S100A12, and S100A8) and other pathways showed a higher pathway expression pattern in lesional AD compared to healthy skin and non-lesional AD skin (Figure 3A–D and Figure S6). Interestingly, multiple genes were involved in the same pathways (Supplementary Figure S7), highlighting that targeted research on these pathways may enhance our understanding and lead to new therapies for lesional and non-lesional AD. Additionally, we performed Pearson correlation analysis between the pathway expression levels and the disease severity measured by the SCORAD and EASI indices to identify pathway expression associated with the disease severity index. The results showed that the pathway expression signatures, including wound healing, glucocorticoid receptor, and S100 family signaling (Figure 4A–F and Supplementary Figure S8), were correlated with SCORAD and EASI.

### 3.6. Validation of Pathways and Gene Signatures Discriminating Lesional, Non-Lesional AD and Healthy Skin

**Figure 5:**
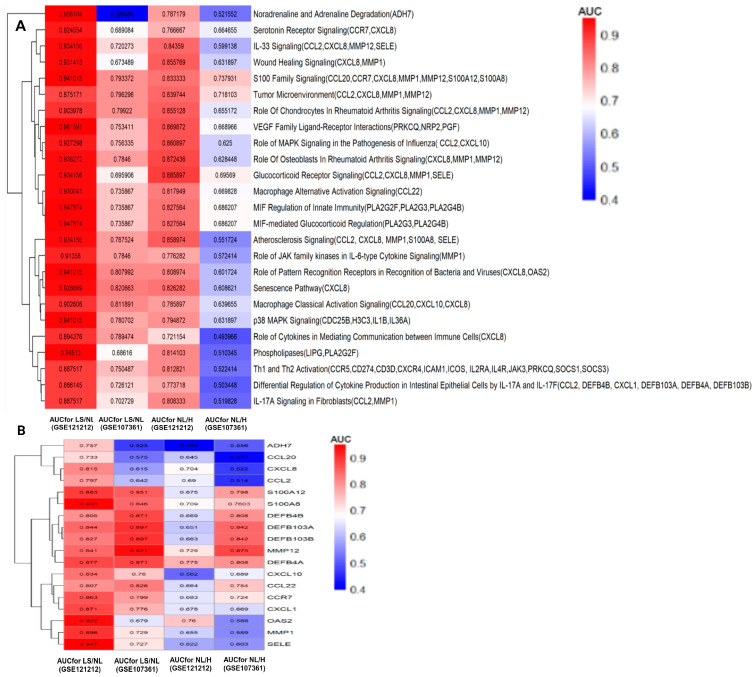
The diagnostic performance of the pathways and gene signatures. (**A**) The AUC values of the pathway signatures with selected representative annotated genes in discriminating lesional AD vs. healthy skin, lesional AD vs. non-lesional AD skin, and non-lesional AD and healthy skin in the discovery and validation datasets. (**B**) The AUC value of the gene signatures in discriminating lesional AD vs. healthy skin, lesional AD vs. non-lesional AD skin, and non-lesional AD and healthy skin in the discovery and validation datasets. LS-lesional skin, NL-non-lesional, H-healthy skin. GSE121212-discovey dataset, GSE107361-validation dataset.

After identifying the relevant pathways and gene signatures that differentiate lesional, non-lesional and healthy skin, we examined the diagnostic performance of the pathways in the discovery and validation datasets (Figure 5A). There were 33 key genes involved in the AD severity-associated pathways, of which 18 genes showed high diagnostic performance in discriminating lesional AD vs. non-lesional AD as well as non-lesional AD vs. health skin, as shown Figure 5B. More specifically, the glucocorticoid receptor and IL-33 signaling-related gene SELE showed high diagnostic efficacy in the GSE121212 (AUC = 0.947) and GSE107361 (AUC = 0.727) datasets between lesional AD vs. non-lesional AD. The atherosclerosis and S100 family signaling-related gene S100A8 showed high diagnostic ability in the GSE121212 (AUC = 0.933), and GSE107361 (AUC = 0.846) datasets between lesional and non-lesional AD. The gene OAS2, related to the role of pattern recognition receptors in recognizing bacteria and viruses, had high diagnostic performance in the GSE121212 (AUC = 0.922) and GSE107361 (AUC = 0.679) datasets between lesional and non-lesional AD. The gene MMP12, associated with multiple pathways such as wound healing, glucocorticoid receptor, and IL-33 signaling, demonstrated high diagnostic ability in discriminating lesional and non-lesional AD in the GSE121212 (AUC = 0.892) and GSE107361 (AUC = 0.729) datasets. Moreover, differential regulation of cytokine production in intestinal epithelial cells by the IL-17A and IL-17F and IL-13 signaling- associated gene DEFB4A had high diagnostic performance in the GSE121212 (AUC = 0.775) and GSE107361 (AUC = 0.808) datasets, followed by OAS2 in the GSE121212 (AUC = 0.76) and GSE107361 (AUC = 0.588) and MMP1 in the GSE121212 (AUC = 0.729) and GSE107361 (AUC = 0.875) datasets in classifying non-lesional AD from healthy skin. Overall, integrative analysis using differential analysis, co-expression networks, and pathway expression ranking via ML approaches identified relevant pathways and gene signatures that discriminate between lesional, non-lesional, and healthy skin.

## 4. Discussion

In the transcriptome-wide pairwise comparison of lesional, non-lesional and healthy skin by integrating differential expression analysis and WGCNA, we identified 975, 441, and 40 DCEGs in lesional vs. healthy skin, lesional vs. non-lesional, and non-lesional vs. healthy skin, respectively, highlighting several clusters of densely interconnected genes involved in lesional skin relative to non-lesional and healthy skin. These DCEGs were enriched in several significant pathways, of which RF prioritized and validated 10 pathway expression signatures, including wound healing (CXCL8 and MMP1), glucocorticoid receptor signaling (CCL2, CXCL8, MMP1and SELE), and S100 family signaling (CXCL8, MMP1 and MMP12), that differentiate lesional, non-lesional, and healthy skin. The pathway expression signatures were correlated with clinical severity measures, including the EASI and SCORAD. Overall, our study demonstrates that high throughput integrative transcriptomics analysis differentiates lesional and non-lesional skin from heathy skin.

Identifying pathways and gene signatures that differentiate lesional, non-lesional, and healthy skin is crucial for AD therapeutics. Previous studies have used differential gene expression analysis to differentiate lesional and non-lesional AD [30,31]. A recent study by Martínez et al. developed an ML method to discriminate between lesional and non-lesional inflammatory skin diseases, including AD, using expression data [10]. Another study by Zhong et al. employed the LASSO method to discover three biomarkers that differentiate non-lesional and lesional AD [18]. However, few studies used combinatorial approaches, including differential analysis, co-expression network analysis, and pathway expression analysis-based on an ML method, to identify pathways and gene signatures that discriminate lesional, non-lesional AD, and healthy skin. These methods can uncover connection networks and hidden biological models crucial to disease pathogenesis [32]. Constructing networks of co-expressed genes identifies highly correlated modules, associates them with phenotypic traits, and elucidates complex gene interactions [33]. This integrative approach demonstrates robustness against noise and variability, offering profound insights into gene regulation and potential therapeutic targets [34].

Therefore, in this study, we used a combinatorial approach, including differential analysis, co-expression network analysis, and machine learning based on pathway expression, to help us select relevant pathways and gene signatures using RNA-seq data from diseased lesional, non-lesional, and healthy skin. More fundamentally, integrated approaches to identify relevant pathway signatures that can predict AD disease severity are crucial. Univariate statistical tests, such as the empirical Bayes-moderated t-statistics test, treat genes as independent from one another and are often poorly reproducible in independent datasets [35]. Therefore, our study used more comprehensive methods to identify the DCEGs in lesional and non-lesional AD, showed these DCEGs were enriched in pathways and gene signatures that discriminated lesional, non-lesional and healthy skin. The 975 DCEGs in lesional vs. healthy, 441 DCEGs in non-lesional vs. healthy, and 40 DCEGs in non-lesional vs. healthy skin were enriched in 108 pathways. Using the RF machine learning algorithm, we showed that the pathway expression levels of 25 identified potential pathways clearly distinguished lesional, non-lesional and healthy skin. Validation analysis confirmed that ten pathway expressions, including wound-healing signaling, glucocorticoid receptor signaling, macrophage alternative activation signaling, IL-33 signaling, S100 family signaling, MIF-mediated glucocorticoid regulation, serotonin receptor signaling, MIF regulation of innate immunity, VEGF family ligand–receptor interactions and tumor microenvironment, showed significant differences among lesional, non-lesional and healthy skin. Remarkably, this study validated that the ten pathway signatures are correlated with the disease severity, including the EASI and SCORAD indices. This may suggest that these pathway trajectories contribute to the progression from non-lesional to lesional AD skin disease.

After identifying pathways associated with lesional and non-lesional skin, we examined the diagnostic relevance of individual genes involved in these pathways. Most of the genes, including MMP12, DEFB103A/B, DEFB4A/B, S100A12, S100A8, CCL22, CCR7, CXCL1, CXCL10, MMP1 and SELE, in these pathways showed high diagnostic efficacy in discriminating lesional from non-lesional skin. In addition, genes such as DEFB4A, OAS2, MMP12 and S100A8 showed high diagnostic performance in classifying non-lesional and healthy samples with an AUC > 0.70 in both discovery and validated datasets. Dysregulated pathways such as the S100 family, tumor microenvironment and IL-33 signaling that contain MMP12 with high diagnostic ability may have functional relevance in AD’s pathology. The wound-healing-related gene signatures such as CXCL8 and MMP1 that were up-regulated in lesional AD compared with non-lesional and healthy skin showed high diagnostic performance in distinguishing lesional AD from non-lesional AD and healthy skin. Interestingly, previous studies indicated that wound healing is a complex process that involves the interaction between different cell types, growth hormones, cytokines, antioxidants, and a stable supply of metal ions [36], and our identified genes may have functional relevance in this complex AD pathology. We also identified three genes, including MMP12, DEFB4A, and S100A8, with high diagnostic value in discriminating lesional AD vs. non-lesional AD as well as non-lesional AD vs. healthy skin. Overall, several genes with high diagnostic value in discriminating lesional, non-lesional and healthy skin might be involved in the disease pathogenesis and could be used as potential biomarkers for the diagnosis of AD and as therapeutic targets of AD. Despite the mechanisms that underlie AD progression still being unclear, in this study, we identified several DCEGs associated with lesional and non-lesional AD skin, and several of the identified genes showed a consistent direction of regulation in lesional and non-lesional AD compared with heathy skin, which were also consistent with previous studies. For example, a previous study identified MMP12 as a potential biomarker of both lesional and non-lesional AD skin [37,38]. A recent study by Guttman-Yassky et al. revealed that a 12-week treatment with abrocitinib, a Janus kinase 1-selective inhibitor, down-regulated the expression of several gene biomarkers, including MMP12 and S100A8, in patients with moderate-to-severe AD skin associated with inflammation, epidermal hyperplasia, and Th2 and Th22 immune responses, highlighting that genes found in our study have potential as biomarkers for monitoring and managing AD disease progression [39]. In addition, another study showed that genes encoding epidermal components such as DEFB4A are up-regulated in lesional skin compared with non-lesional AD skin [14]. Although there are numerous reports of gene expression in healthy skin, less is known about the architecture of clinically uninvolved non-lesional skin as compared to healthy skin. Examination of non-lesional skin in AD could provide previously unknown insights into the molecular processes operating in uninvolved skin and suggest a unique preclinical set of healthy skin in non-lesional condition. Non-lesional skin may be more useful in identifying the driving features underlying pathogenesis because the inflammatory milieu among diseases becomes more similar during the lesional stage. Our study indicated that non-lesional skin samples are extremely informative about the underlying disease process and could be used to create patient subsets for future clinical trials or de-risk clinical trials, as non-lesional skin is reported to be an effective marker of the treatment response [40].

Our study has several strengths. Integrated analysis of the correlation network analysis and ML method identified disease-relevant pathways such as MIF regulation of innate immunity and IL-33 signaling, inflammatory-related pathway–wound healing and metabolic-related pathways–serotonin signaling, which can be used to distinguish lesional from non-lesional and healthy skin. Pathway prioritization in discriminating lesional, nonregional and healthy skin based on the ML approach showed little similarity to pathway prioritization based on the traditional method, which indicates that ML-based pathway prioritization may provide an alternative approach to identify relevant pathways associated with the disease phenotype. This study highlights pathways correlated with disease activity, suggesting their relevance to disease pathogenesis. However, the study faces limitations such as the small sample sizes in the gene expression datasets and the absence of detailed clinical variables. The relatively small sample sizes in some public datasets may hinder the identification of potential association pathways and gene signatures related to AD severity. Future research should address these limitations by increasing the sample sizes, incorporating clinical factors, and conducting functional experiments to validate the findings.

## 5. Conclusions

In summary, we presented evidence supporting the predictive roles of pathway and gene expression signatures in distinguishing between lesional and non-lesional AD through an integrative analysis of networks and pathway expressions based on a machine learning approach. Notably, the pathway signatures were closely correlated with the AD severity measures, including the EASI and SCORAD indices. These findings may have practical implications for clinical practice, as these signatures could enhance the accuracy of predicting AD skin involvement, thereby informing more effective treatment strategies for AD disease.

## Data Availability

All the transcriptomics datasets used in our study are freely accessible at NCBI GEO (https://www.ncbi.nlm.nih.gov/geo/) with accession numbers GSE121212, GSE107361 and GSE130588, accessed on 5 March 2024.

## Supplementary Materials

The following supporting information can be downloaded at https://www.mdpi.com/article/10.3390/jpm14090960/s1.
