# Supplementary material for: Co-Expression Network and Machine Learning Analysis of Transcriptomics Data Identifies Distinct Gene Signatures and Pathways in Lesional and Non-Lesional Atopic Dermatitis
Source: J Pers Med. 2024 Sep 10;14(9):960. doi: 10.3390/jpm14090960 (PMC11433539; doi:10.3390/jpm14090960)
Supplement: Supplementary file 1 [file jpm-14-00960-s001.zip › Suplementary Table S1.pdf]

Co-Expression Network and Machine Learning Analysis of Transcriptomics Data Identifies Distinct Gene Signatures and Pathways in Lesional and Non-Lesional Atopic Dermatitis

Eskezeia Y. Dessie [1], Lili Ding [2], Latha Satish [1] and Tesfaye B. Mersha [1,*]

[1]Division of Asthma Research, Cincinnati Children's Hospital Medical Center, Department of Pediatrics, University of Cincinnati College of Medicine, 3333 Burnet Avenue, Cincinnati, OH 45229-3039, USA, eskezeia.dessie@cchmc.org (E.Y.D.); latha.satish@cchmc.org (L.S.); tesfaye.mersha@cchmc.org

[2]Division of Biostatistics and Epidemiology, Cincinnati Children's Hospital Medical Center, Department of Pediatrics, University of Cincinnati College of Medicine, 3333 Burnet Avenue, Cincinnati, OH, 45229-3039, USA; lili.ding@cchmc.org

* Correspondence: tesfaye.mersha@cchmc.org; Tel.: +1 513-803-2766; Fax: +1 513-636-1657

Supplementary Table S1. The gene expression datasets used in our study.

GEO ID	Sample size Lesional, /non-lesional/healthy	Gender (% female)	Age(years) mean ±SD	EASI	SCORAD	Platform
GSE121212	27/27/38	NA	NA	NA	NA	Illumina HiSeq 2500
GSE107361	39/40/29	40	23.5±24.8	NA	NA	Affymetrix Human Genome U133 Plus 2.0 Array
GSE130588	51/42/20 (week 0)	48	NA	31.7±13.8	62.76±15.4	Affymetrix Human Genome U133 Plus 2.0 Array

In the GSE121212 discovery dataset- there were a total of 147 samples including 27 lesional AD, 27 non-lesional AD, and 38 control skin, 28 lesional PSO, and 27 non-lesional PSO with RNA-seq gene expression profiles. Since our study focused on atopic dermatitis, we removed lesional skin of psoriasis (PSO)- 28 lesional PSO, 27 non-lesional PSO. Hence, we used 27 lesional AD, 27 non-lesional AD, and 38 control skin samples as discovery dataset. In the GSE107361 dataset, there were a total of 39 healthy skin, 40 non-lesional AD and 29 lesional AD skin samples with microarray gene expression profiles. This dataset was used as validation for the finding obtained from discovery dataset. In the GSE130588 another validation dataset, there were a total of 208 samples with microarray gene expression profiles. This dataset consisting of longitudinal data collected at different weeks including at week 0 (baseline data) having 51 lesional AD, 42 non-lesional AD, and 20 heathy skin samples; at week 4, there were 44 lesional AD samples only, and at week16, there were 29 lesional AD, and 22 non-lesional AD. Since the samples

received treatment with Dupilumab at week 4 and week 16, and hence treatment may affect the gene expression profiles, therefore, in this study we used the data collected at baseline :51 lesional AD, 42 non-lesional AD, and 20 heathy skin along with AD severity measures including To examine the pathway expression association between disease severity index measures including EASI and SCORAD, we used baseline samples having lesional and non-lesional samples with corresponding AD severity index. NA-denote-not available.
