# Supplementary material for: Co-Expression Network and Machine Learning Analysis of Transcriptomics Data Identifies Distinct Gene Signatures and Pathways in Lesional and Non-Lesional Atopic Dermatitis
Source: J Pers Med. 2024 Sep 10;14(9):960. doi: 10.3390/jpm14090960 (PMC11433539; doi:10.3390/jpm14090960)
Supplement: Supplementary file 1 [file jpm-14-00960-s001.zip › Suplemetary Figures S1-S8.pdf]

Co-Expression Network and Machine Learning Analysis of Transcriptomics Data Identifies Distinct Gene Signatures and Pathways in Lesional and Non-Lesional Atopic Dermatitis

Eskezeia Y. Dessie [1], Lili Ding [2], Latha Satish [1] and Tesfaye B. Mersha [1,*]

[1]Division of Asthma Research, Cincinnati Children's Hospital Medical Center, Department of Pediatrics, University of Cincinnati College of Medicine, 3333 Burnet Avenue, Cincinnati, OH 45229-3039, USA, eskezeia.dessie@cchmc.org (E.Y.D.); latha.satish@cchmc.org (L.S.); tesfaye.mersha@cchmc.org

[2]Division of Biostatistics and Epidemiology, Cincinnati Children's Hospital Medical Center, Department of Pediatrics, University of Cincinnati College of Medicine, 3333 Burnet Avenue, Cincinnati, OH, 45229-3039, USA; lili.ding@cchmc.org

* Correspondence: tesfaye.mersha@cchmc.org; Tel.: +1 513-803-2766; Fax: +1 513-636-1657

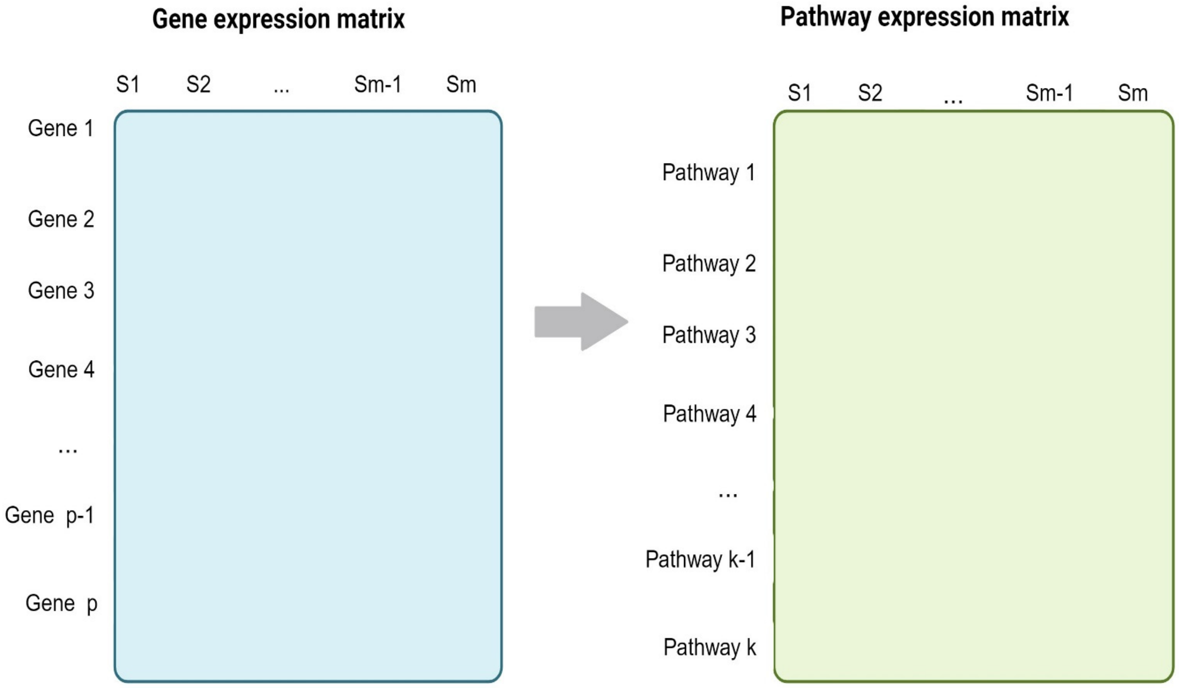

Supplementary Figure S1: Transformation gene expression data matrix into pathway expression data matrix. Let $X \epsilon R^{p \times m}$ be gene expression matrix having p gene expression levels for m samples. We transform X to pathway expression matrix m having k pathway expression levels for p samples. Let $P(i,j)$ be pathway transition matrix/pathway membership with the *i*th row and *j*th column of m. $P(i,j) = \begin{cases} cj(i) & if\ gene\ i\ is\ in\ pathway\ j \\ 0 & \end{cases} = \begin{cases} 1 & if\ gene\ i\ is\ in\ pathway\ j \\ 0 & \end{cases}$

Then, we transform pathway average expression as follow $Y_j = \frac{\sum_{i=1}^{m} p(i,j) X_{i.}}{\sum_{i=1}^{p} p(i,j)}$

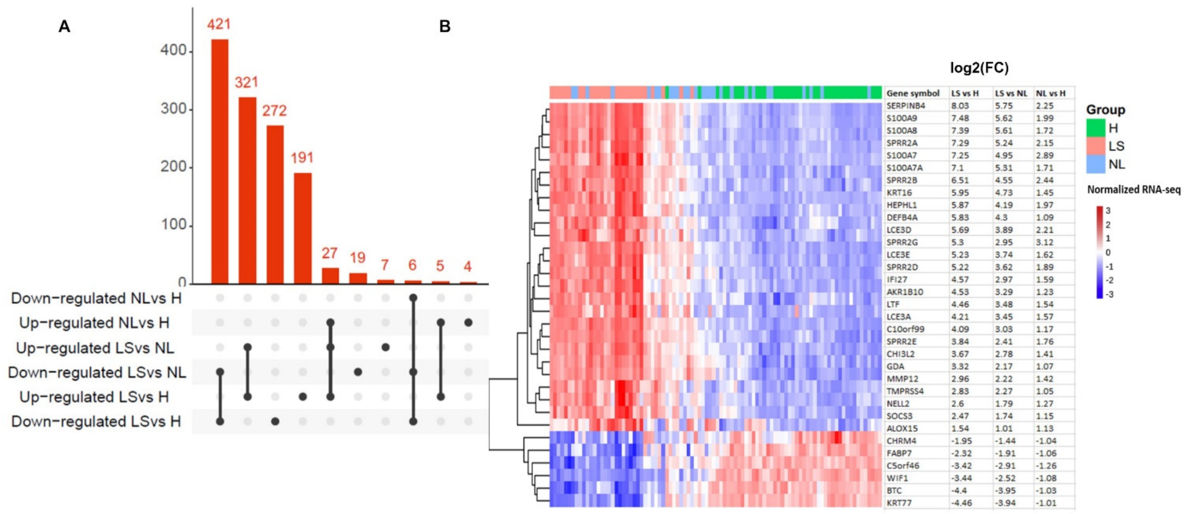

Gene symbol	LS vs H	LS vs NL	NL vs H
SERPINB4	8.03	5.75	2.25
S100A9	7.48	5.62	1.99
S100A8	7.39	5.61	1.72
SPRR2A	7.29	5.24	2.15
S100A7	7.25	4.95	2.89
S100A7A	7.1	5.31	1.71
SPRR2B	6.51	4.55	2.44
KRT16	5.95	4.73	1.45
HEPHL1	5.87	4.19	1.97
DEFB4A	5.83	4.3	1.09
LCE3D	5.69	3.89	2.21
SPRR2G	5.3	2.95	3.12
LCE3E	5.23	3.74	1.62
SPRR2D	5.22	3.62	1.89
IFI27	4.57	2.97	1.59
AKR1B10	4.53	3.29	1.23
LTF	4.46	3.48	1.54
LCE3A	4.21	3.45	1.57
C10orf99	4.09	3.03	1.17
SPRR2E	3.84	2.41	1.76
CHI3L2	3.67	2.78	1.41
GDA	3.32	2.17	1.07
MMP12	2.96	2.22	1.42
TMPRSS4	2.83	2.27	1.05
NELL2	2.6	1.79	1.27
SOCS3	2.47	1.74	1.15
ALOX15	1.54	1.01	1.13
CHRM4	-1.95	-1.44	-1.04
FABP7	-2.32	-1.91	-1.06
C5orf46	-3.42	-2.91	-1.26
WIF1	-3.44	-2.52	-1.08
BTC	-4.4	-3.95	-1.03
KRT77	-4.46	-3.94	-1.01

Supplementary Figure S2: Identification of common differentially expressed genes (DEGs) DEGs across three comparison groups: A) Upset plot showing common and unique DEGs in lesional AD, non-lesional AD and healthy skin. B) heatmap showing the clustering of lesional AD, non-lesional AD and healthy skin based on 33 common DEGs. LS-lesional skin, NL- non-lesional, and H- healthy skin.

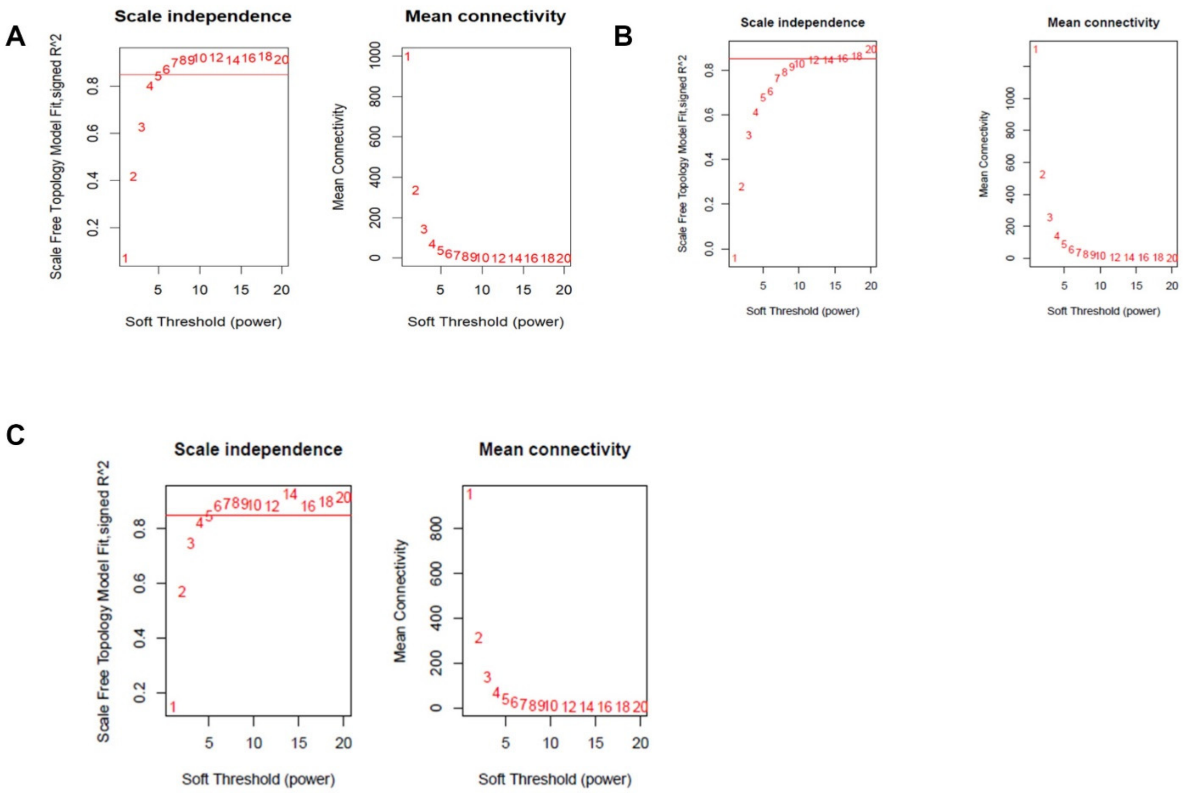

Supplementary Figure S3: WGCNA analyses to select soft thresholding power for A) LS vs H comparisons B) LS vs NL comparisons C) NL vs H comparisons. For LS vs H comparisons, WGCNA was constructed using the suitable soft threshold power (β) = 5 (scale free topology model fit signed topology R2 = 0.85. For LS vs NL comparison data, WGCNA was constructed using the suitable soft threshold power (β) = 10 (scale free topology model fit signed topology R2 = 0.85). For NL vs H comparison data, WGCNA was constructed using the suitable soft threshold power (β) = 5 (scale free topology model fit signed topology R2 = 0.85). LS-lesional skin, NL- non-lesional, and H- healthy skin.

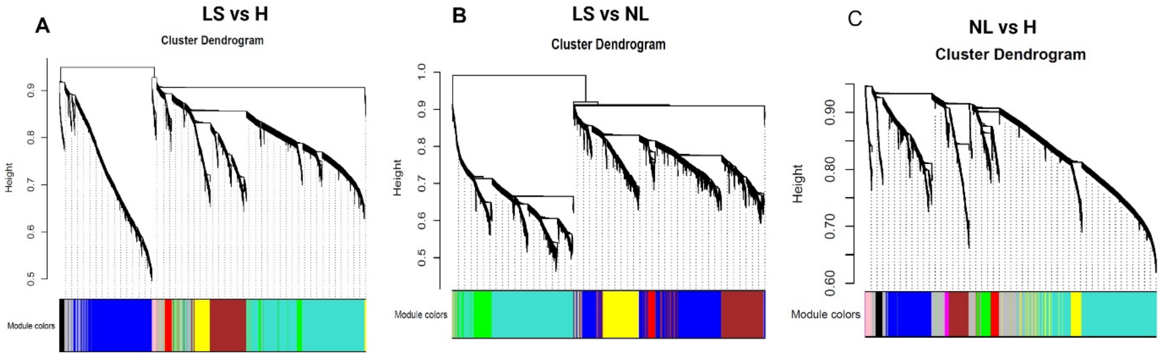

Supplementary Figure S4: Clustering of 4905 hypervariable genes in A) lesional vs healthy, B) lesional vs non-lesional, C) non-lesional and healthy comparisons. There was a total of 9 co-expression modules in lesional vs healthy, 12 co-expression modules in lesional vs non-lesional, and 10 co-expression modules non-lesional and healthy comparisons. LS-lesional skin, NL- non-lesional, and H- healthy skin.

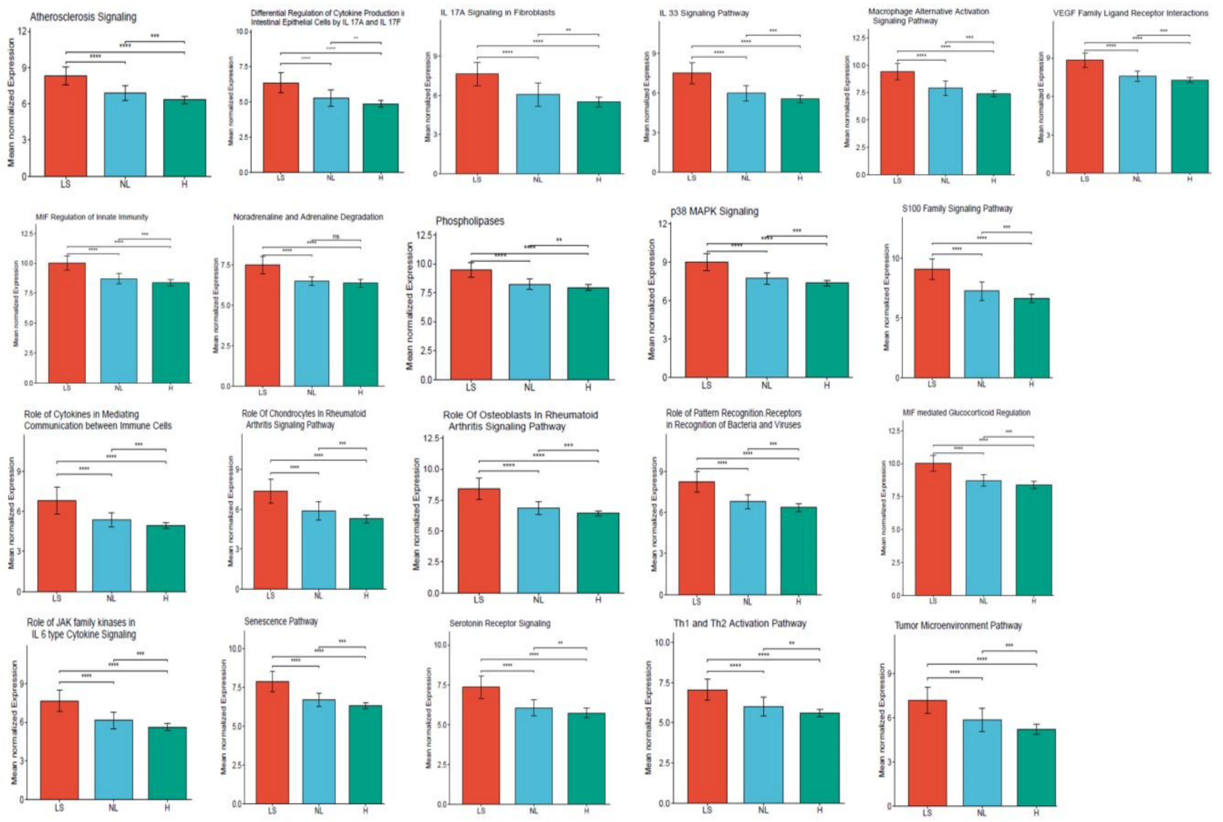

Supplementary Figure S5: Comparison of different pathways expression in lesional AD, non-lesional AD and control skin in discovery GSE121212 dataset. The mean normalized expression of annotated genes for each pathway in lesional, non-lesional and healthy skin were compared using t-test to identify differential pathway signature between groups. LS-lesional skin, NL- non-lesional, and H- healthy skin.

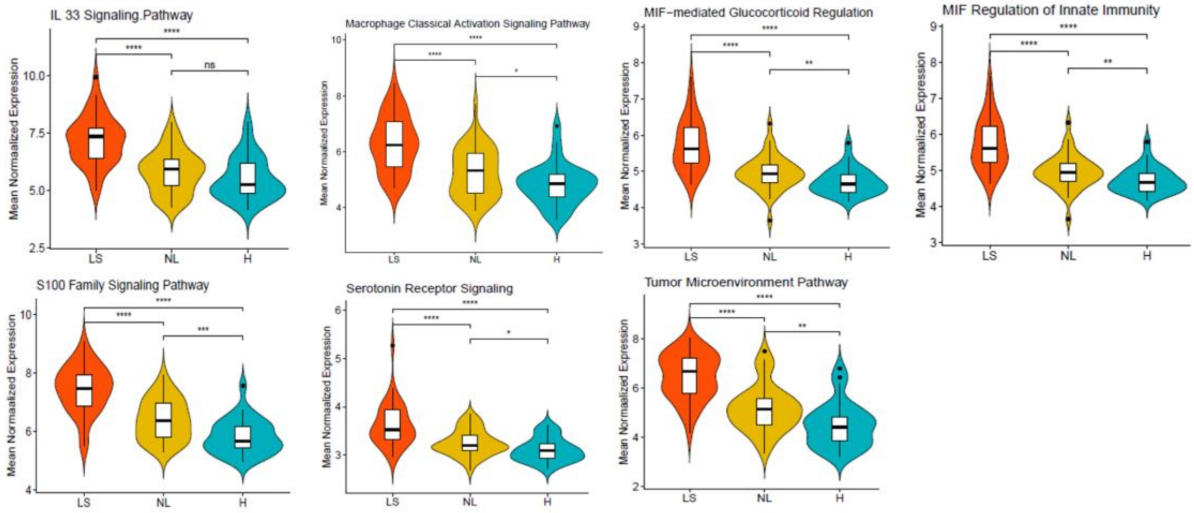

Supplementary Figure S6: Comparison of different pathways expression in lesional AD, non-lesional AD and control skin in validation GSE107361 dataset. The mean normalized expression of annotated genes for each pathway in lesional, non-lesional and healthy skin were compared using t-test to identify differential pathway signature between groups. LS-lesional skin, NL- non-lesional, and H- healthy skin.

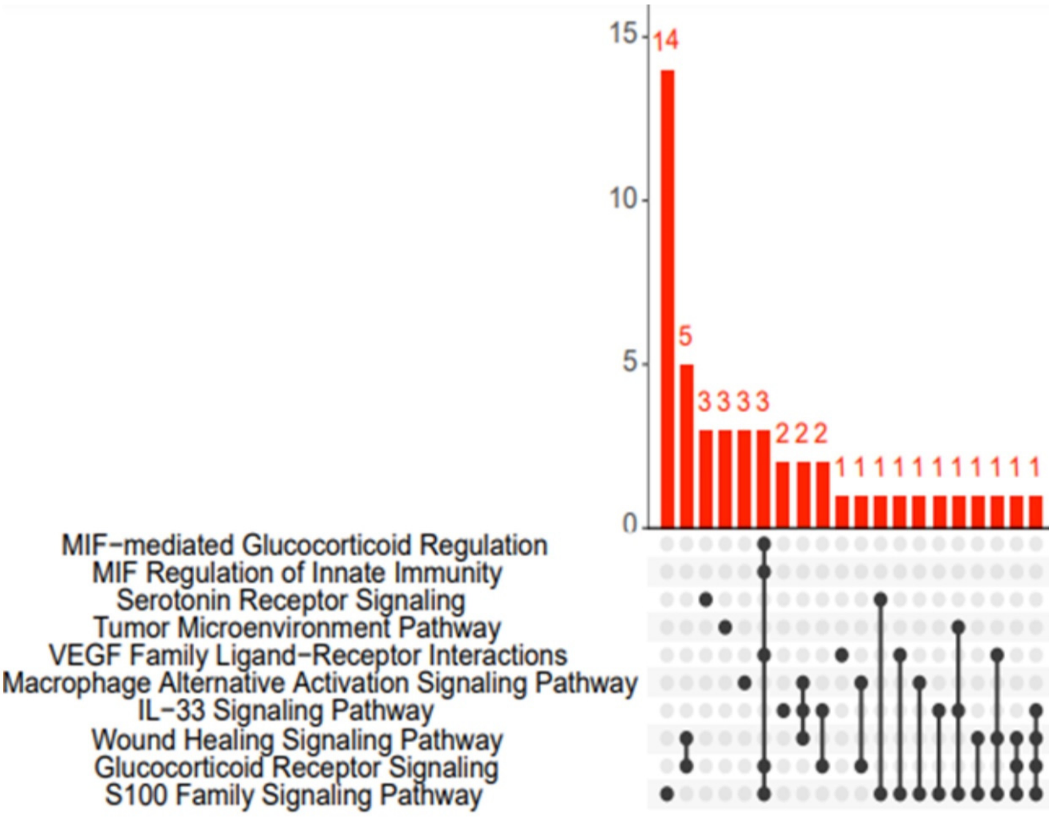

Supplementary Figure S7: Upset plot showing the numbers of common and unique difeerentially co-expressed genes-DCEGs involved in lesional AD, non-lesional AD and healthy skin discrimninatriy pathways.

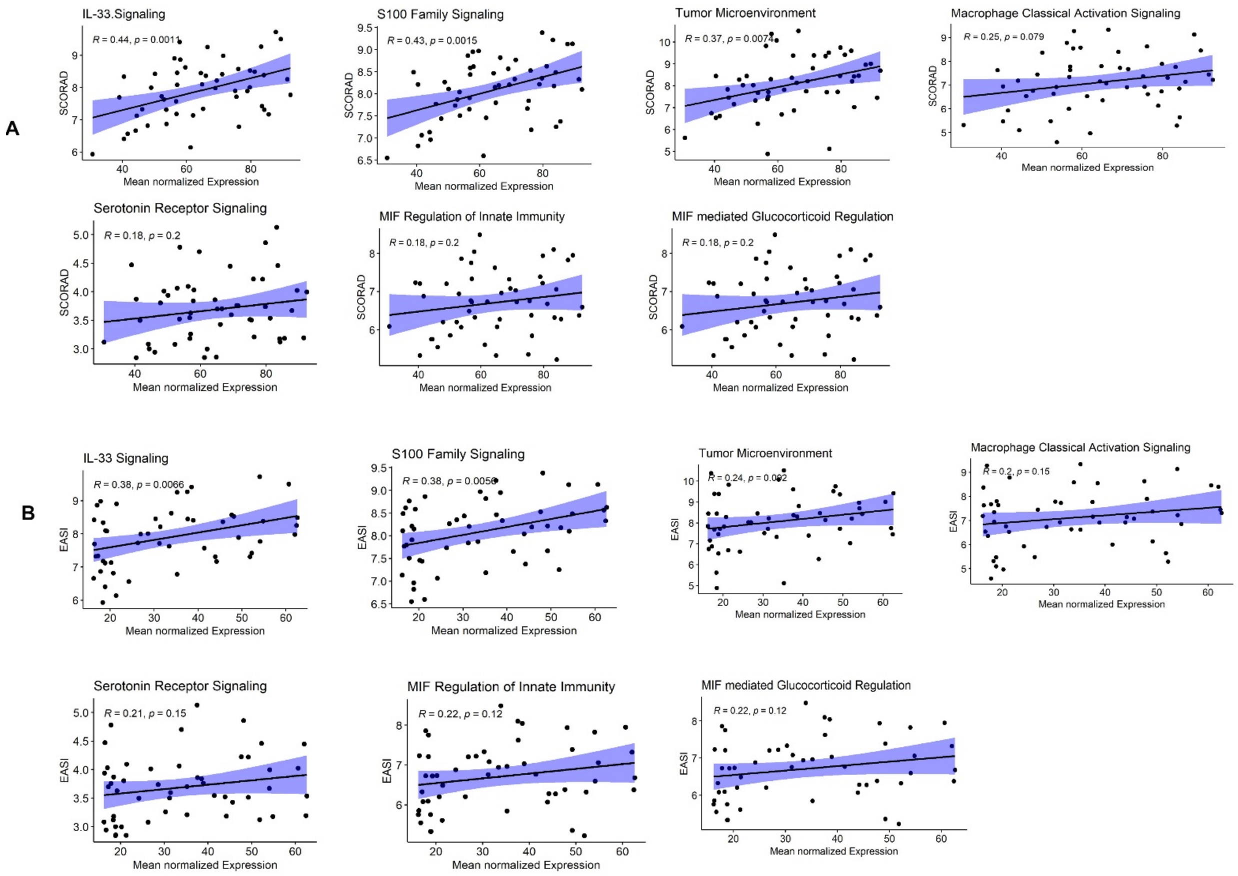

Supplementary Figure S8: Association of pathway mean expression levels and disease severity measure: SCORAD and EASI index in lesional and non-lesional AD skin samples in validation dataset (data collected at week0-baseline study in the GSE130588 dataset.
